# STRA6 regulates tumor immune microenvironment and is a prognostic marker in BRAF-mutant papillary thyroid carcinoma

**DOI:** 10.3389/fendo.2023.1076640

**Published:** 2023-02-10

**Authors:** Weiman He, Yijia Sun, Jiawei Ge, Xuejie Wang, Bo Lin, Shuang Yu, Yanbing Li, Shubin Hong, Haipeng Xiao

**Affiliations:** ^1^ Department of Endocrinology, The First Affiliated Hospital of Sun Yat-sen University, Guangzhou, China; ^2^ Department of Thyroid Surgery, The First Affiliated Hospital of Sun Yat-sen University, Guangzhou, China

**Keywords:** papillary thyroid carcinoma (PTC), BRAF mutation, STRA6, prognosis, tumor immune microenvironment

## Abstract

**Background:**

BRAF mutation is one of the most common genetic alterations contributing to the initiation and progression of papillary thyroid carcinoma (PTC). However, the prognostic value of BRAF mutation for PTC is limited. Novel markers are needed to identify BRAF-mutant patients with poor prognosis.

**Methods:**

Transcriptional expression data were downloaded from the Cancer Genome Atlas (TCGA) and Gene Expression Omnibus (GEO) datasets. Pathway enrichment was performed by Kyoto Encyclopedia of Genes and Genomes (KEGG) analysis and gene set enrichment analysis (GSEA). Protein-protein interaction networks were predicted by the GeneMANIA. The correlation between STRA6 expression and immune infiltration was analyzed by tumor immune estimation resource (TIMER) and tumor-immune system interaction database (TISIDB). Immunohistochemistry was used to detect the STRA6 protein expression level of PTC. Infiltration of regulatory T cells (Tregs) and CD8+ T cells in tumor samples were analyzed by fluorescent multiplex immunohistochemistry.

**Results:**

In BRAF-mutant PTC, STRA6 was extremely upregulated and predicted unfavorable survival, which was an independent risk factor for increased mortality risk. Bioinformatic analyses indicated that STRA6 might activate the MAPK pathway synergistically with BRAF^V600E^. The expression of STRA6 was associated with immune infiltrates and T cell exhaustion. Fluorescent multiplex immunohistochemistry showed that STRA6 increased Tregs abundance and decreased CD8+ T cells infiltration in PTC. Moreover, STRA6 promoted epithelial-mesenchymal transition *via* increased cancer-associated fibroblasts infiltration.

**Conclusions:**

Our study demonstrates STRA6 may serve as a prognostic marker for BRAF-mutated PTC, which may drive thyroid carcinogenesis *via* activation of oncogenic pathway and regulation of tumor immunosuppressive microenvironment.

## Introduction

Thyroid carcinoma (TC) is the most common endocrine malignancy with an increasing incidence worldwide (1). Most of TCs arise from thyroid follicular epithelial cells, among which 80% are papillary thyroid carcinoma (PTC) (2). Initiation and progression of TC involve multiple genetic alterations, of which the most common oncogenic mutation is BRAF^V600E^ (3, 4). Although BRAF^V600E^ mutation is associated with poorer recurrence-free probability (5), it was suggested that not all of the PTCs with BRAF^V600E^ mutation belonged to high-risk recurrence in American Thyroid Association Management Guidelines (6) or even were associated with aggressive clinicopathological outcomes (7, 8). Thus, novel markers are needed to identify BRAF-mutant PTC patients with poor prognosis.

STRA6, functions as a Vitamin A transporter and cytokine receptor (9), has been reported in multiple cancers. STRA6 activated the Wnt/β-catenin signaling in gastric cancer and served as a prognostic biomarker (10, 11). Upregulation of STRA6 in colon cancer was correlated with poor oncologic prognosis and transduced JAK2-STAT signaling, which contributes to colon tumorigenesis (12, 13). Moreover, STRA6 polymorphisms were correlated with clinical characteristics and might be potential markers in locally-advanced and metastatic non-small cell lung cancer (14). However, the prognostic value of STRA6 in PTC remains unknown.

Tumor microenvironment, composed of tumor cells, immune cells, cytokines, etc., plays an essential role in cancer metastasis, progression and recurrence. The escape of tumor cells often occurs in an immunosuppressive microenvironment (15). It was reported that regulatory T cells (Tregs) were enriched in primary thyroid tumors (16) and had higher infiltration in lymph node metastases (17). Besides, the density of tumor-associated macrophages was increased in advanced thyroid cancer, which correlated with decreased survival rate of patients (18). Recent study revealed that STRA6 was associated with infiltration of antigen-presenting cells in stomach adenocarcinoma, which could serve as a potential mRNA vaccine candidate (19). Nevertheless, the engagement of STRA6 in tumor immune microenvironment of TC remains elusive.

In this study, we demonstrate STRA6 is upregulated in BRAF-mutant PTC. High STRA6 expression is associated with unfavorable prognosis for individuals with BRAF^V600E^. Immune infiltration and pathway enrichment were investigated in samples with high STRA6 expression. STRA6 may contribute to the progression of BRAF-mutant PTC *via* regulation of immunosuppressive tumor microenvironment and activation of oncogenic pathway.

## Materials and methods

### Transcriptional data

Transcriptional expression data and clinicopathological information of the Cancer Genome Atlas (TCGA) cohort were downloaded from UCSC Xena (https://xena.ucsc.edu/). GSE199023 was downloaded from Gene Expression Omnibus (GEO) datasets (https://www.ncbi.nlm.nih.gov/). The RNA sequencing data of the FAH-SYSU cohort was obtained from our previous study (20).

### Tumor immune estimation

Tumor immune estimation resource (TIMER) 2.0 (http://timer.cistrome.org/) is a web server for comprehensive analysis of immune infiltrates with diverse algorithms. It was applied to analyze STRA6 expression level in pan-cancer based on the TCGA dataset. The correlation of STRA6 expression between BRAF mutation status was further explored by Timer 2.0. It was also used to analyze the correlation of immune infiltrates with STRA6 expression and BRAF mutation status. The relation between immunomodulators and STRA6 expression was evaluated *via* tumor-immune system interaction database (TISIDB) (http://cis.hku.hk/TISIDB/index.php).

### Differential expression analysis and functional annotations

NetworkAnalyst **(** https://www.networkanalyst.ca/) is a platform for comprehensive gene expression profiling analysis. We divided the BRAF-mutant PTC from the TCGA cohort into two groups according to STRA6 expression. After uploading a gene expression table, differently expressed genes (DEGs) were analyzed by the platform and followed with Kyoto Encyclopedia of Genes and Genomes (KEGG) analysis. Additionally, principal component analysis (PCA) between two groups was performed on Clustvis website (https://biit.cs.ut.ee/clustvis/). Protein-protein interaction (PPI) was predicted by GeneMANIA (http://genemania.org/).

### Gene set enrichment analysis

GSEA software was downloaded from the website (http://www.gsea-msigdb.org/gsea/index.jsp). We performed analysis in 50 patients with high STRA6 expression and 50 patients with low STRA6 expression of the TCGA cohort. Hallmark gene sets were used to explore the mechanism of STRA6 in thyroid carcinoma progression.

### Immunohistochemistry

The study was approved by the Institutional Research Ethics Committee of The First Affiliated Hospital of Sun Yat-sen University (no. [2021] 109). 30 pairs of PTC tissues were collected from the First Affiliated Hospital of Sun Yat-sen University with informed consent from patients. Paraffin embedded tissues were deparaffinized with xylene for two times and dehydrated with a series of ethanol. Antigen retrieval was performed by boiling the slides in EDTA buffer (C1034, Solarbio). After cooling, the slides were blocked with 20% goat serum at room temperature for 30 min and incubated with STRA6 primary antibody (diluted 1:200, H00064220-D01P, Novus) at 4°C overnight. Followed by rinse with PBS for 3 times, the slices were incubated with secondary antibody at room temperature for 30 min. Detection of protein expression was performed with a 3,3’-diaminobenzidine (DAB) peroxidase substrate kit (K5007; Dako) under a microscope. Subsequently, the slides were counterstained with Harris hematoxylin, followed by dehydration in graded alcohol and incubation in xylene. The immunohistochemistry (IHC) staining score was calculated with the following formula: IHC score = extent score × intensity score. The staining extent was scored as 0 (1%–4% positive cells), 1(5%–25% positive cells), 2 (26%–50% positive cells), 3 (51%–75% positive cells), 4 (≥75% positive cells). The staining intensity was scored as 0-3, corresponding to no staining, weak staining, intermediate staining and strong staining, respectively.

### Fluorescent multiplex immunohistochemistry

Fluorescent multiplex immunohistochemistry (mIHC) was applied to characterize immune infiltration in PTC. After incubation with primary antibody, the slides were incubated with an HRP-conjugated secondary antibody and fluorophore-conjugated Tyramide Signal Amplification reagent (Cat. 10003100100, Panovue). Multiplex panel design was as follows: FoxP3 (diluted 1:100, ab215206, abcam), Opal 570 TSA (diluted 1:200); CD8 (diluted 1:100, 70306S, CST), Opal 690 TSA (diluted 1:200). Finally, the slides were stained with DAPI (4’, 6-diamidino-2-phenylindole) and mounted with anti-fated reagent.

### Statistical analysis

Experimental data was analyzed with GraphPad Prism 8.0 and presented as mean ± standard deviation (SD). Comparison between two groups was analyzed with Mann-Whitney U test or Student’s t test. The correlation between STRA6 expression and clinicopathological features in the TCGA cohort was analyzed with a chi-squared test. Kaplan–Meier analysis with a log-rank test was used to evaluate the prognostic value of STRA6 in BRAF-mutant PTC. Univariate Cox regression analysis was performed to investigate the correlation between variables and survival. After multivariate adjustment for age, gender, tumor size, multifocality, extrathyroidal extension and lymph node metastasis, multivariate Cox regression analysis further accessed whether high STRA6 expression was an independent risk factor for recurrence and mortality. A *P* value of <0.05 was considered statistically significant.

## Results

### STRA6 is upregulated in BRAF-mutated PTC.

**Figure 1:**
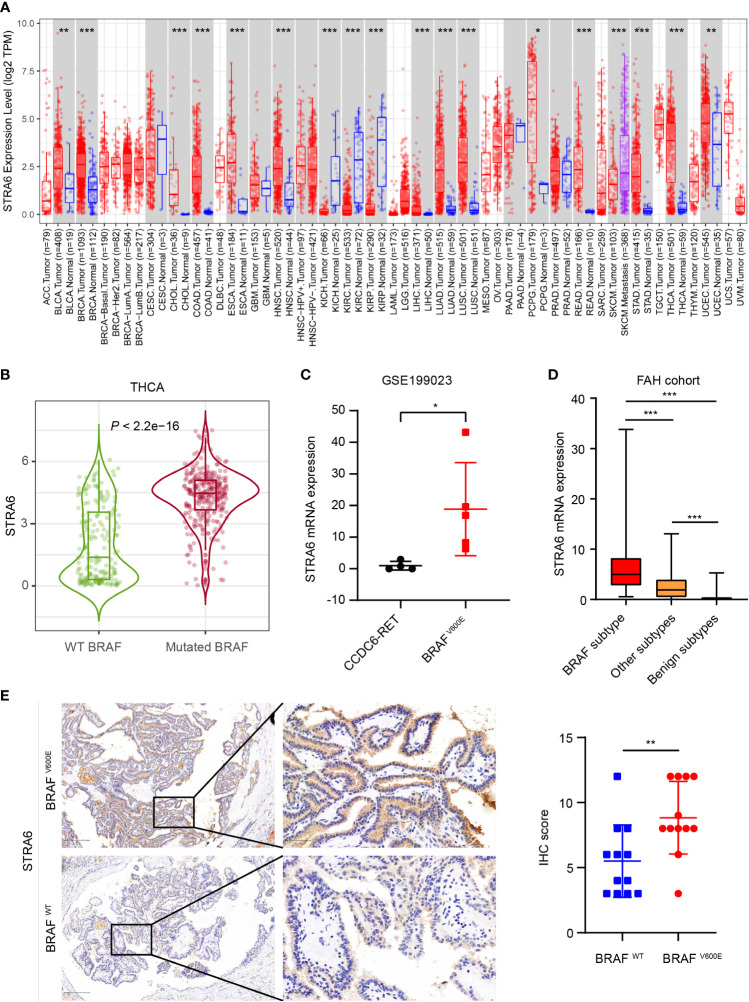
STRA6 is upregulated in BRAF-mutated PTC. **(A)** mRNA expression of STRA6 in various cancers according to the TCGA cohort. **(B)** STRA6 mRNA expression based on BRAF genotype in THCA cohort. **(C)** STRA6 mRNA expression of zebrafish thyroid tumors with oncogenic transfection gene in GSE199023. **(D)** mRNA abundance in FAH cohort with respect to different molecular subtypes. **(E)** Immunohistochemistry (IHC) staining of STRA6 protein level based on the BRAF mutation status in PTC samples. (*p < 0.05, **p < 0.01, ***p < 0.001. The data are shown as the mean ± SD).

To unveil the expression pattern of STRA6 across different cancers, we analyzed the mRNA expression level of STRA6 in the TCGA cohort. STRA6 was upregulated in most cancers including thyroid carcinoma (**Figure 1A**). Notably, the expression of STRA6 was positively correlated with BRAF mutation in TC and melanoma (**Figure S1A**). Compared with BRAF wild-type (WT) PTC, STRA6 was significantly upregulated in BRAF-mutated samples (**Figure 1B**). Based on a public dataset GSE199023, zebrafish thyroid tumors transfected with BRAF^V600E^ oncogenes showed higher expression of STRA6 as compared to those transfected with CCDC6-RET fusion (**Figure 1C**). We further analyzed the mRNA expression in our cohort based on distinct molecular subtypes. STRA6 was remarkably upregulated in BRAF subtype compared with other PTC subtypes and benign nodules (**Figure 1D**). Overexpression of STRA6 protein level in BRAF^V600E^ PTC was also confirmed by IHC (**Figure 1E**). Taken together, STRA6 is upregulated in thyroid cancer and exhibits extremely higher expression in BRAF-mutant PTC.

### Upregulation of STRA6 predicts aggressive outcomes and unfavorable prognosis for BRAF-mutant PTC

**Figure 2:**
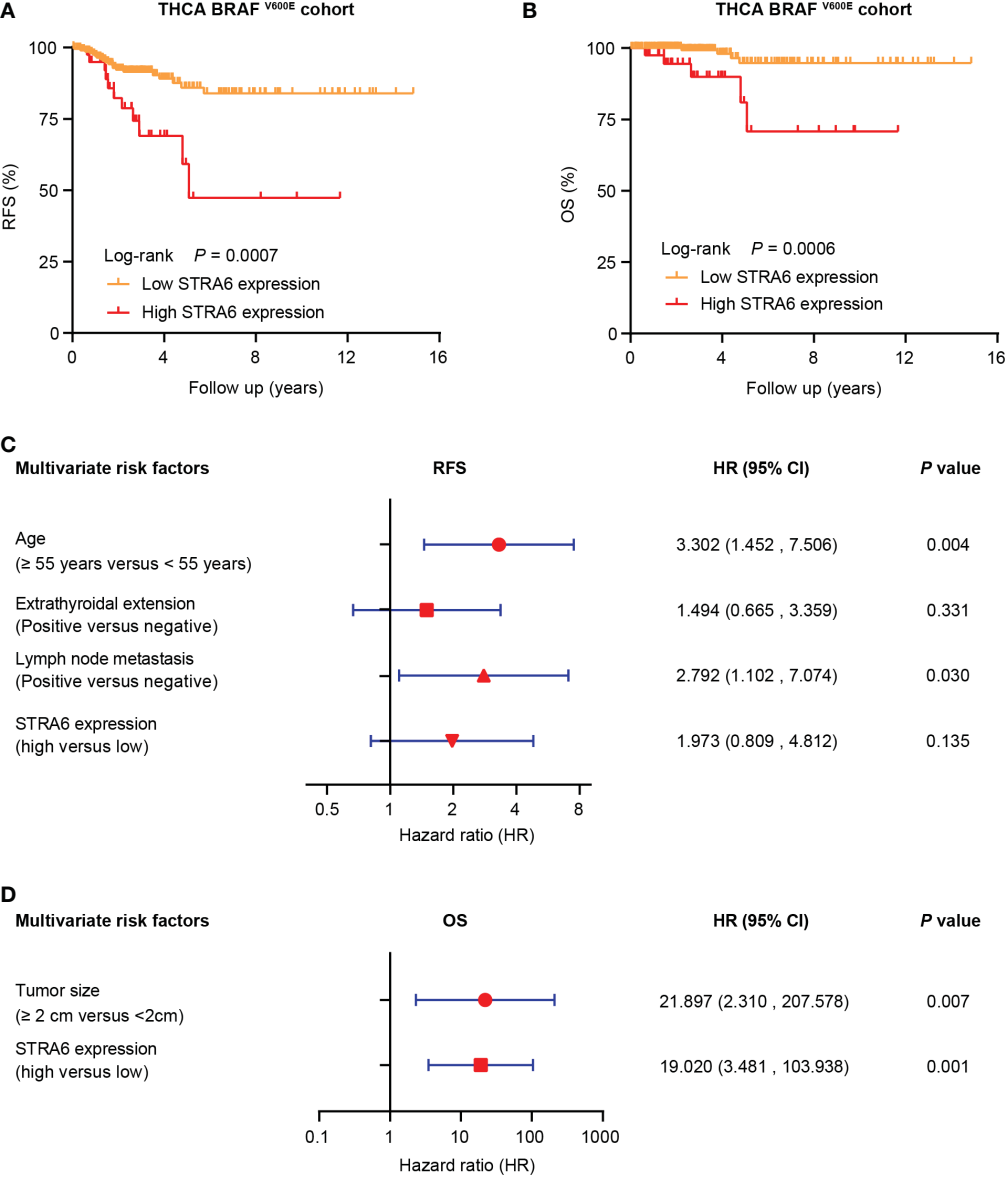
STRA6 predicts poor prognosis for BRAF-mutant PTC. **(A)** Kaplan–Meier survival curves of recurrence-fee survival (RFS) according to STRA6 expression in BRAF-mutant PTC. Prognostic cutoff value was identified as 10.3 using X-Tile. **(B)** Kaplan-Meier analysis of overall survival (OS) based on STRA6 expression in PTC patients with BRAF mutation. **(C)** Multivariate Cox regression analysis of the risk factors for recurrence in BRAF-mutant PTC. All bars represented 95% confidence intervals (95% CI). **(D)** Multivariate Cox regression analysis of the risk factors for mortality in BRAF-mutant PTC. All bars corresponded to 95% CI.

We investigated the relationship between STRA6 expression and clinicopathological features in the TCGA cohort. High STRA6 expression was associated with BRAF mutation (*P* = 0.001) and distant metastasis (*P* = 0.019) (**Table 1**). Then we divided PTC patients into two groups according to the genotype of BRAF. Intriguingly, high STRA6 expression was significantly associated with aggressive clinicopathological outcomes in BRAF-mutant PTC, but not in BRAF WT groups. For individuals with BRAF mutation, rates of distant metastasis were 2/130 (1.5%) vs. 3/26 (11.5%) (*P* = 0.033) in low-STRA6 group vs. high-STRA6 group, respectively. The recurrence rates were significantly higher in high-STRA6 PTC than low-STRA6 PTC [11/40 (27.5%) vs. 20/212 (9.4%), *P* = 0.003]. As for mortality, it was robustly higher in PTC with high STRA6 expression than those with low STRA6 expression [5/40 (12.5%) vs. 4/212 (1.9%), *P* = 0.004] (**Table 2**). Kaplan-Meier analysis showed higher STRA6 expression significantly predicted poorer recurrence free survival (RFS) (*P* = 0.0007) and overall survival (OS) (*P* = 0.0006) (**Figures 2A, B**).

**Table 1:** The association between STRA6 expression and clinicopathologic characteristics in the TCGA cohort.

	Total	Low-STRA6	High-STRA6	*P* value
	n (%)	n (%)	n (%)	
Age ≥55 years	167/504 (33.1%)	142/447 (31.8%)	25/57 (43.9%)	0.068
Sex (female)	368/504 (73.0%)	330/447 (73.8%)	38/57 (66.7%)	0.251
Multifocality	227/494 (46.0%)	202/438 (46.1%)	25/56 (44.6%)	0.835
Tumor size (≥2.0 cm)	151/406 (37.2%)	130/356 (36.5%)	21/50 (42.0%)	0.453
Extrathyroidal extension	153/486 (31.5%)	131/430 (30.5%)	22/56 (39.3%)	0.181
BRAF mutation	252/495 (50.9%)	212/439 (48.3%)	40/56 (71.4%)	0.001
Lymph node metastasis	224/454 (49.3%)	195/402 (48.5%)	29/52 (55.8%)	0.324
Distant metastasis	9/291 (3.1%)	5/253 (2.0%)	4/38 (10.5%)	0.019^a^
Stage (III/IV)	166/502 (33.1%)	141/445 (31.7%)	25/57 (43.9%)	0.066

a Continuity Correction.

**Table 2:** Correlation between STRA6 expression and clinicopathological characteristics of PTC in the TCGA cohort based on the BRAF genotypes.

	Wild-type BRAF			BRAF V600E mutation		
	Low-STRA6	High-STRA6	*P* value	Low-STRA6	High-STRA6	*P* value
Age ≥55 years	76/227 (33.5%)	6/16 (37.5%)	0.742	63/212 (29.7%)	18/40 (45.0%)	0.058
Sex (female)	168/227 (74.0%)	10/16 (62.5%)	0.476^a^	157/212 (74.1%)	27/40 (67.5%)	0.392
Multifocality	103/220 (46.8%)	5/16 (31.3%)	0.227	94/210 (44.8%)	20/39 (51.3%)	0.453
Tumor size (≥2.0 cm)	67/177 (37.9%)	7/13 (53.8%)	0.254	60/171 (35.1%)	13/36 (36.1%)	0.907
Extrathyroidal extension	48/216 (22.2%)	7/16 (43.8%)	0.099^a^	81/207 (39.1%)	14/39 (35.9%)	0.704
Lymph node metastasis	90/202 (44.6%)	9/16 (56.3%)	0.366	105/195 (53.8%)	19/35 (54.3%)	0.962
Distant metastasis	3/118 (2.5%)	1/11 (9.1%)	0.303^b^	2/130 (1.5%)	3/26 (11.5%)	0.033^b^
Stage (III/IV)	65/226 (28.8%)	4/16 (25.0%)	0.972^a^	72/211 (34.1%)	20/40 (50.0%)	0.056
Recurrence	18/227 (7.9%)	3/16 (18.8%)	0.304^a^	20/212 (9.4%)	11/40 (27.5%)	0.003^a^
Mortality	6/227 (2.6%)	1/16 (6.3%)	0.383^b^	4/212 (1.9%)	5/40 (12.5%)	0.004^a^

a Continuity Correction.

b Fisher’s Exact Test.

### High STRA6 expression is an independent risk factor for mortality in BRAF-mutated PTC

Furthermore, we explored the hazard ratios (HRs) of STRA6-related risk (**Table 3**). In the entire TCGA cohort, univariate Cox regression analysis showed that upregulation of STRA6 was an independent risk factor for recurrence and mortality, with HRs being 2.98 (1.62-5.48) (*P* < 0.001) and 4.67 (1.69-12.87) (*P* = 0.003), respectively. After multivariate adjustment for age, gender, tumor size, multifocality, extrathyroidal extension and lymph node metastasis, HRs of recurrence and mortality remained significantly (*P* = 0.013 and *P* = 0.017). Then we divided patients into two groups according to BRAF mutation status. In BRAF wild-type patients, neither recurrence rates nor mortality rates was associated with STRA6 expression. In BRAF-mutant patients, STRA6-related recurrence risk was 3.32 (1.59-6.96) (*P* =0.001) and became 1.97 (0.81-4.81) (*P* = 0.135) after multivariate adjustment (**Figure 2C**). STRA6 overexpression was a risk factor for mortality with an HR being 7.12 (1.91-26.56) (*P* = 0.003), which became more significant with an HR being 19.02 (3.48-103.94) (*P* = 0.001) after multivariate adjustment (**Figure 2D**). Therefore, individuals with high STRA6 expression show increased mortality risk in BRAF-mutated PTC.

**Table 3:** Hazard ratios of high STRA6 expression vs low STRA6 expression in risk of recurrence and mortality in the THCA cohort with respect to BRAF status.

	HR (95%CI)	*P* value	Adjusted HR (95%CI)	*P* value
Entire TCGA cohort				
Recurrence	2.98 (1.62, 5.48)	<0.001	2.39 (1.20, 4.76)	0.013
Mortality	4.67 (1.69, 12.87)	0.003	4.29 (1.30, 14.16)	0.017
Wild-type BRAF subtype				
Recurrence	2.55 (0.75, 8.69)	0.136	–	–
Mortality	2.53 (0.30, 21.37)	0.395	–	–
BRAF V600E subtype				
Recurrence	3.32 (1.59, 6.96)	0.001	1.97 (0.81, 4.81)	0.135
Mortality	7.12 (1.91, 26.56)	0.003	19.02 (3.48, 103.94)	0.001

"-" means not applicable.

### STRA6 plays oncogenic and immunoregulatory roles in BRAF-mutated PTC progression

**Figure 3:**
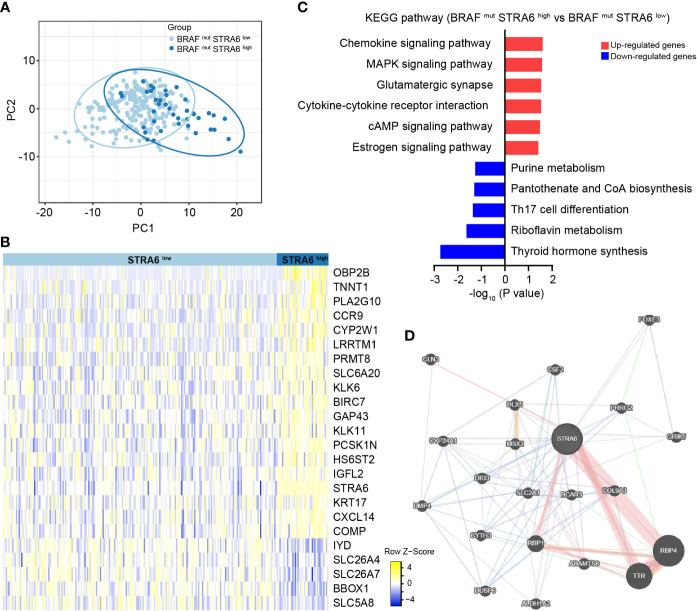
STRA6 plays immunoregulatory and oncogenic roles in BRAF-mutated PTC. **(A)** Principal component analysis (PCA) plot of BRAF-mutated samples based on STRA6 expression. **(B)** Differently expressed genes (DEGs) between high-STRA6 group and low-STRA6 group. **(C)** Kyoto Encyclopedia of Genes and Genomes (KEGG) pathway enrichment analysis based on upregulated genes and downregulated genes. **(D)** Proteins interacted with STRA6 were predicted by GeneMANIA.

To explore the mechanism of STRA6 contributing to the poor prognosis in BRAF-mutant PTC, we divided patients into two groups according to STRA6 expression. Principal component analysis (PCA) showed separation between high-STRA6 PTC and low-STRA6 PTC (**Figure 3A**). Differential expression genes (DEGs) between two groups were shown in **Figure 3B**. Of which, most genes were involved in malignant tumor progression, such as OBP2B, TNNT1, KLK6, KRT17 etc. Moreover, chemokine signaling pathway and cytokine-cytokine receptor interaction were enriched with the upregulated genes in high-STRA6 groups, indicating STRA6 may engage in the regulation of the immune interaction (**Figure 3C**). We also found that STRA6 expression was associated with immunomodulators in the TCGA cohort (**Figures S1B, C**), which indicated that STRA6 may function as an immunoregulator in TC progression. Additionally, the MAPK signaling pathway, which has emerged as one of the frequently activated pathways in thyroid tumorigenesis, was also remarkably enriched with the upregulated genes in high-STRA6 PTC. Downregulation of genes enriched in thyroid hormone synthesis further suggested the oncogenic role of STRA6 in TC progression (**Figure 3C**). PPI networks of STRA6 were constructed by GeneMANIA. It was predicted that 20 proteins were binding to STRA6. Of note, STRA6 interacted with several proteins modulated by the MAPK signaling pathway, including DIO3, BMP4 and DUSP6 (**Figure 3D**). Therefore, our results demonstrate that STRA6 may contribute to BRAF-mutated PTC progression by regulating oncogenic pathways and immune microenvironment.

### STRA6 is correlated with immune infiltration in thyroid carcinoma

**Figure 4:**
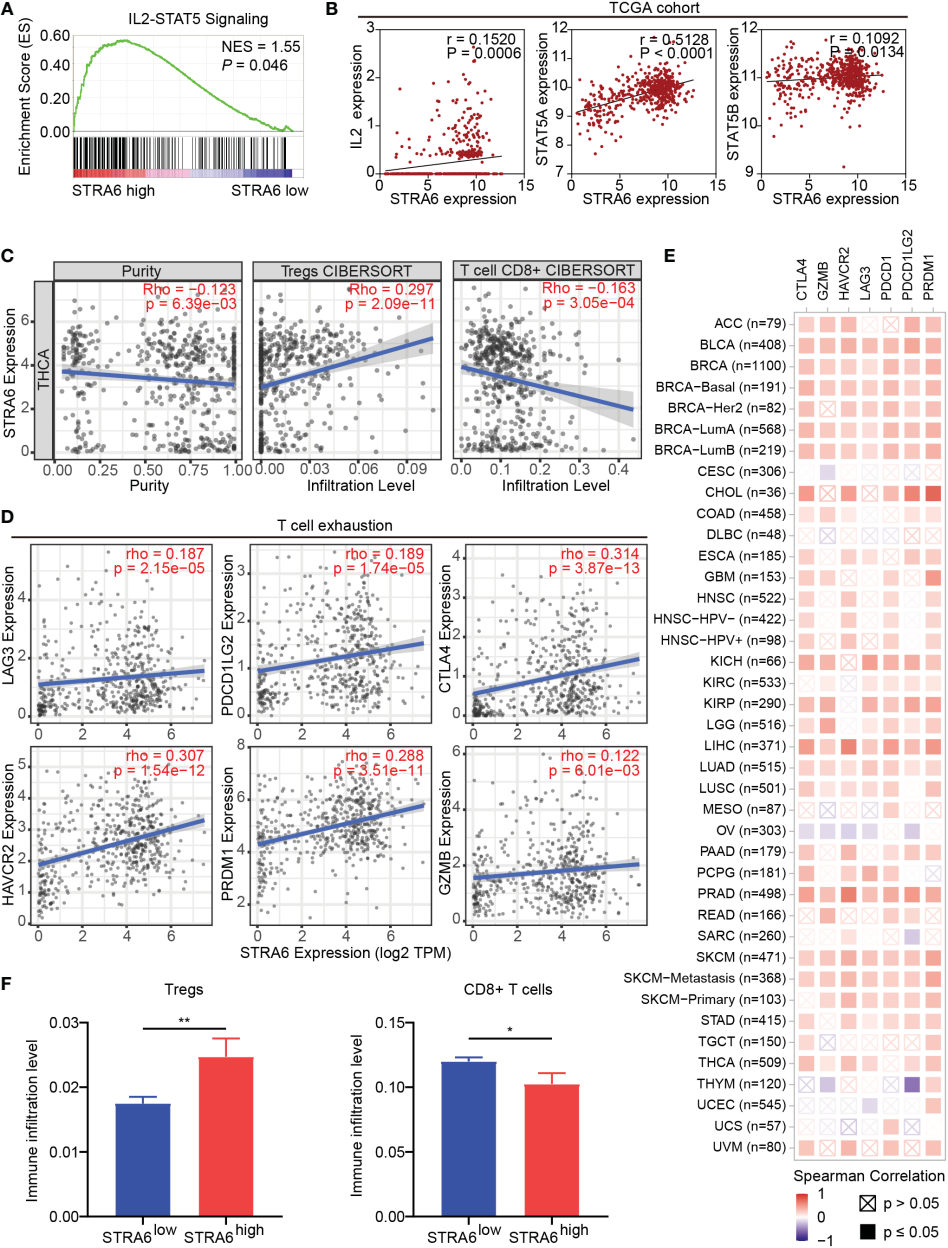
STRA6 regulates immunosuppressive microenvironment *via* the IL2-STAT5 signaling pathway. **(A)** Gene set enrichment analysis (GSEA) of the IL2-STAT5 signaling pathway enriched in samples with high-STRA6 expression. **(B)** Correlation of the expression of STRA6 and key molecules in the IL2-STAT5 pathway. **(C)** The correlation of STRA6 expression with infiltration of Tregs and CD8+ T cells according to the TCGA datasets. **(D)** Scatterplots of correlations between STRA6 expression and T cell exhaustion markers in the TCGA cohort. **(E)** Spearman correlations between STRA6 expression and T cell exhaustion markers in pan-cancer. **(F)** Immune infiltration level of Tregs and CD8+ T cells in PTC based on STRA6 expression. (*p < 0.05, **p < 0.01. The data are shown as the mean ± SEM).

Given immune infiltration takes part in tumor progression, we performed Gene Set Enrichment Analysis (GSEA) with transcriptome data from the TCGA cohort. The results showed that samples with high STRA6 expression enriched in the IL2-STAT5 signaling pathway (**Figure 4A**). STRA6 expression was positively correlated with the mRNA level of IL2, STAT5a and STAT5b (**Figure 4B**). IL2 is a critical immunomodulatory cytokine, which increases Tregs and induces CD8+ T cell exhaustion *via* activation of STAT5. Thus, we evaluated the association between STRA6 expression and immune cell infiltration in PTC samples. After purity adjustment, upregulation of STRA6 was correlated with abundance of Tregs. However, STRA6 expression was negatively correlated with the infiltration of CD8+ T cells (**Figure 4C**). We also assessed the expression of exhausted T cells markers, including LAG3, PDCD1G2, CTLA4, HAVCR2, PRDM1 and GZMB in the TCGA cohort. STRA6 was positively correlated with most T cell exhaustion markers (**Figure 4D**). Pan-caner analysis also revealed the role of STRA6 in inducing T cell exhaustion (**Figure 4E**). Tregs were significantly abundant in high-STRA6 PTC, while CD8+ T cells had a lower infiltration level (**Figure 4F**). The infiltration of other immune cells was also correlated with STRA6 expression, including NK cells, neutrophils, MDSCs, macrophages, myeloid dendritic cells, monocytes and B cells (**Figure S2A**).

**Figure 5:**
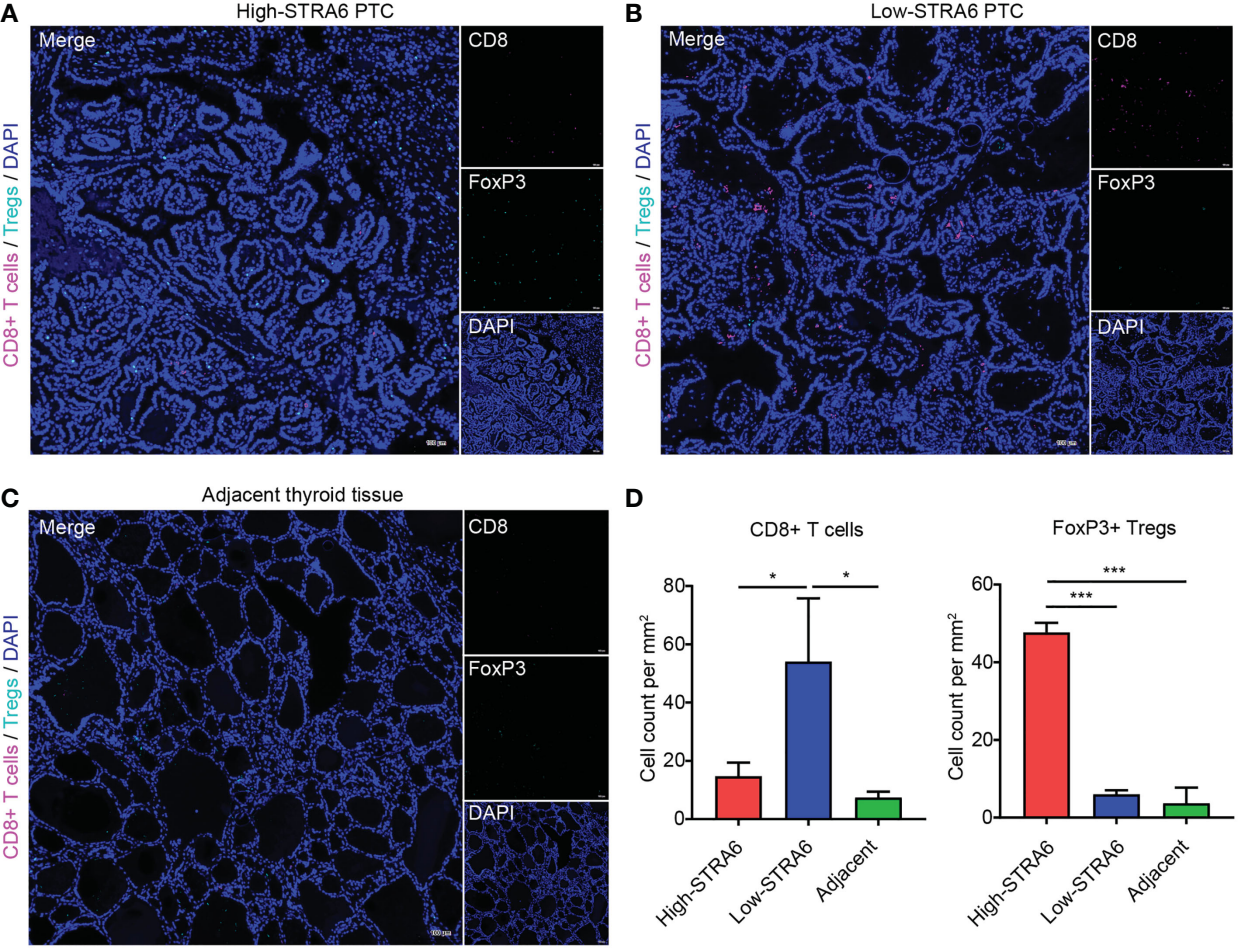
STRA6 increases Tregs abundance and inhibits CD8+ T cells infiltration in PTC. **(A–C)** Representative images of fluorescent multiplex immunohistochemistry (mIHC) in high-STRA6 PTC, low-STRA6 PTC and adjacent thyroid tissue. CD8+ T cells were stained with CD8α and Tregs were stained with FoxP3. Nuclei were stained with DAPI. **(D)** Positive Tregs and CD8+ T cells were counted in each group. (*p < 0.05, ***p < 0.001. The data are shown as the mean ± SD).

To confirm the role of STRA6 in Tregs and CD8+ T cells infiltration, we performed fluorescent multiplex immunohistochemistry (mIHC) in PTC with different STRA6 expression. Samples with high STRA6 expression were mainly infiltrated with FoxP3+ Tregs while CD8+ T cells were mainly observed in samples with low STRA6 expression (**Figures 5A, B**). Adjacent thyroid tissues were less infiltrated with immune cells (**Figure 5C**). Upregulation of STRA6 was associated with lower CD8+ cells and higher Tregs infiltrates (**Figure 5D**), which was consistent with the infiltration level of immune cells in PTC with BRAF mutation (**Figures S3A, B**). Therefore, STRA6 may limit antitumor immunity and induce immunosuppressive tumor microenvironment in TC.

### STRA6 promotes epithelial-mesenchymal transition *via* increased cancer-associated fibroblasts infiltration in thyroid carcinoma

**Figure 6:**
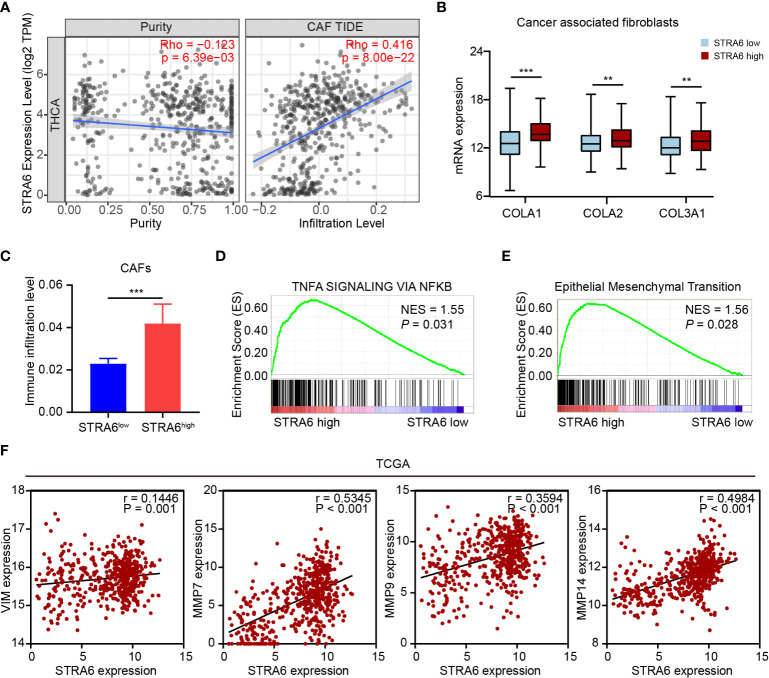
STRA6 promotes EMT process *via* increased CAFs infiltration. **(A)** The correlation of STRA6 expression with CAFs infiltration after purity adjustment. **(B)** mRNA expression of CAFs markers in PTC based on STRA6 expression. **(C)** Infiltration level of CAFs in low-STRA6 PTC and high-STRA6 PTC. **(D, E)** GSEA results of TNFα/NF-κB signaling pathway and epithelial mesenchymal transition enrichment according to the TCGA database. **(F)** Scatter plot analysis of correlation between STRA6 mRNA expression and EMT markers. (**p < 0.01, ***p < 0.001. The data are shown as the mean ± SEM).

(CAF) Cancer-associated fibroblast is one of the important components in tumor microenvironment. CAFs infiltration was positively associated with the expression of STRA6 (**Figure 6A**). We also analyzed the expression level of CAFs markers in PTC. COLA1, COLA2 and COL3A1 were all upregulated in high-STRA6 samples (**Figure 6B**). Compared with low-STRA6 group, CAFs were abundant in high-STRA6 group (**Figure 6C**). Higher infiltration level of CAFs was also observed in BRAF-mutant PTC (**Figure S3C**). CAFs play important roles in cancer development *via* regulation of metastasis. The results of GSEA showed that the TNFα/NF-κB signaling pathway and Epithelial-Mesenchymal Transition (EMT) hallmark gene set was significantly enriched in high-STRA6 PTC (**Figures 6D, E**). Consistently, the mRNA expression of STRA6 was positively correlated with the expression of EMT markers, including Vimentin (r = 0.1446), MMP7 (r = 0.5345), MMP9 (r = 0.3594), MMP14 (r = 0.4984) (**Figure 6F**). Taken together, STRA6 may promote TC metastasis *via* CAF-mediated EMT process.

## Discussion

As the most common genetic alteration in PTC, the prevalence of BRAF mutation differs in races. Compared with western populations (3), the incidence of BRAF mutation was much higher in Chinese population (4, 21), which was detected in about 75% PTC patients. However, BRAF mutation may not be a single and independent prognostic marker for PTC. Studies revealed that BRAF mutation may not totally associate with clinicopathological characteristics or survival (22, 23). In our study, we found STRA6 is associated with BRAF genotype. Upregulation of STRA6 predicts poor RFS and OS in BRAF-mutant PTC, which is an independent risk factor for mortality. Previous study showed that BRAF^V600E^ mutation was significantly correlated with PTC-related mortality in an unadjusted analysis and the association was no longer significant after adjusting for multiple clinical factors (24). Thus, our study indicated that STRA6 may act as a prognostic marker, which may provide individualized diagnosis and therapeutic strategy for BRAF-mutant PTC.

We further explored the mechanism of STRA6 in BRAF-mutant PTC progression. Interestingly, when we divided BRAF-mutant PTC into two groups according to STRA6 expression, the MAPK signaling pathway is significantly enriched with the upregulated genes. STRA6 also interacts with several proteins regulated by the MAPK pathway, including BMP4, DIO3, and DUSP6. It was reported that BMP4 can inhibit heat-induced apoptosis by enhancing the activation of ERK (25). DIO3 is increased in PTC through activation of the MAPK pathway (26, 27). DUSP6 is a member of the MAPK phosphatase family and plays pro-tumorigenic role in TC (28, 29). Moreover, BRAF mutation drives aberrant activation of the MAPK pathway (30) and promotes TC tumorigenesis. Dual activation of the MAPK pathway by STRA6 and BRAF mutation, may contribute to the poor survival of BRAF-mutant PTC with high STRA6 expression.

Pathway analyses showed that STRA6 involves in the regulation of the immune interaction and activation of the IL2-STAT5 pathway. Our study also revealed that STRA6 expression is correlated with the infiltration of multiple immune cells. Notably, upregulation of STRA6 results in higher Tregs abundance and lower CD8+ T cells infiltration. Additionally, STRA6 is correlated with T cell exhaustion. In support of our results, IL-2 is mainly produced by activated CD4+ T cells and induces the expansion of Tregs (31). Persistent activation of the IL2-STAT5 pathway triggers CD8+ T cells into exhausted status (32). Decreased infiltration of CD8+ T cells and a low CD8/regulatory T cell ratio in tumors are critical for unfavorable prognosis of cancers (33, 34). Our results also showed that high STRA6 expression is associated with increased CAFs and promotes EMT process, which further explains the worse prognosis in BRAF-mutant PTC. CAFs are one of the most abundant stromal components in the tumor microenvironment. By secreting TGF-β, CCL2, CCL5, IL-6 etc., CAFs recruit immunosuppressive cells into the tumor stroma, which contributes to cancer metastasis and progression (35). The secretion of various cytokines promotes aggressive phenotypes in tumor cells *via* the activation of EMT process (36). We also confirmed BRAF^V600E^ mutation was associated with suppressive immune cell infiltration (37). In hence, immune suppression mediated by STRA6 and BRAF contributes to PTC progression synergistically.

Taken together, STRA6 is overexpressed in TC and associated with BRAF mutation. Upregulation of STRA6 predicts unfavorable prognosis for BRAF-mutant PTC. High STRA6 expression is correlated with decreased CD8+ T cells as well as abundant Tregs and CAFs, which induces immunosuppressive microenvironment in TC. Moreover, STRA6 may play an oncogenic role *via* the MAPK pathway and EMT process. In conclusion, STRA6 might be a prognostic marker for BRAF-mutant PTC and individualized therapeutic strategies for the patients are needed.

## Data availability statement

The original contributions presented in the study are included in the article/**Supplementary Material**. Further inquiries can be directed to the corresponding authors.

## Ethics statement

The studies involving human participants were reviewed and approved by the Institutional Research Ethics Committee of The First Affiliated Hospital of Sun Yat-sen University. The patients/participants provided their written informed consent to participate in this study.

## Author contributions

HX, SH and WH conceived and designed the study. WH, JG, and XW performed the bioinformatic analysis. WH and YS performed immunohistochemistry and fluorescent multiplex immunohistochemistry. WH analyzed the data and wrote the manuscript with the approval of all other authors. BL collected tissue samples. SY and YL gave critical comments. HX and SH supervised the study. All authors contributed to the article and approved the submitted version.
