# Supplementary material for: STRA6 regulates tumor immune microenvironment and is a prognostic marker in BRAF-mutant papillary thyroid carcinoma
Source: Front Endocrinol (Lausanne). 2023 Feb 10;14:1076640. doi: 10.3389/fendo.2023.1076640 (PMC9950572; doi:10.3389/fendo.2023.1076640)
Supplement: Supplementary file 1 [file DataSheet_1.docx]

Supplementary Material

# Supplementary Figures
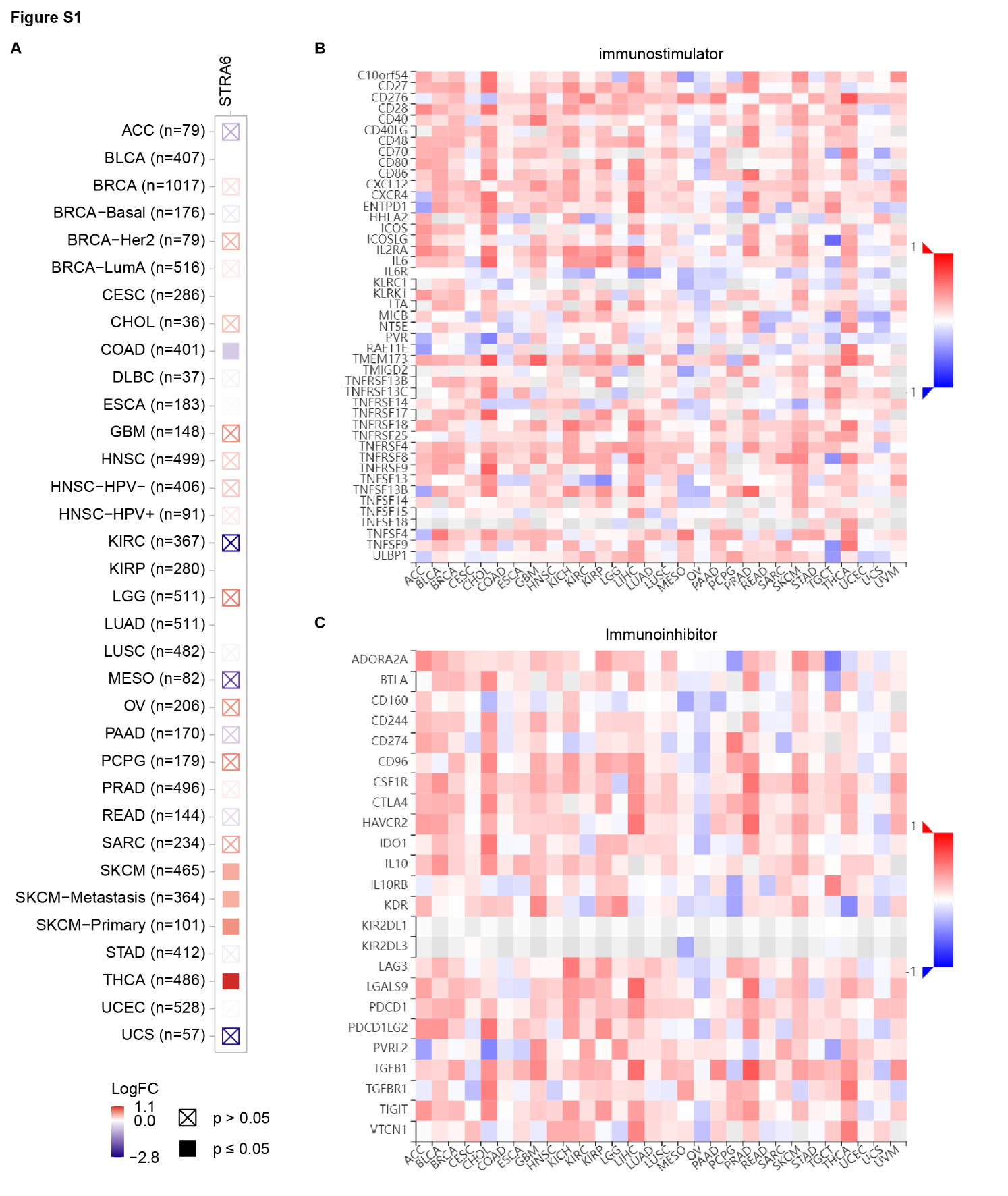


**Supplementary Figure 1.** Upregulation of STRA6 is correlated with BRAF status and immunomodulators in pan-cancers. **(A)** The correlation of STRA6 expression with BRAF mutation in pan-cancer according to TCGA datasets. **(B-C)** The relation between immunomodulators and STRA6 expression was evaluated by TISIDB.
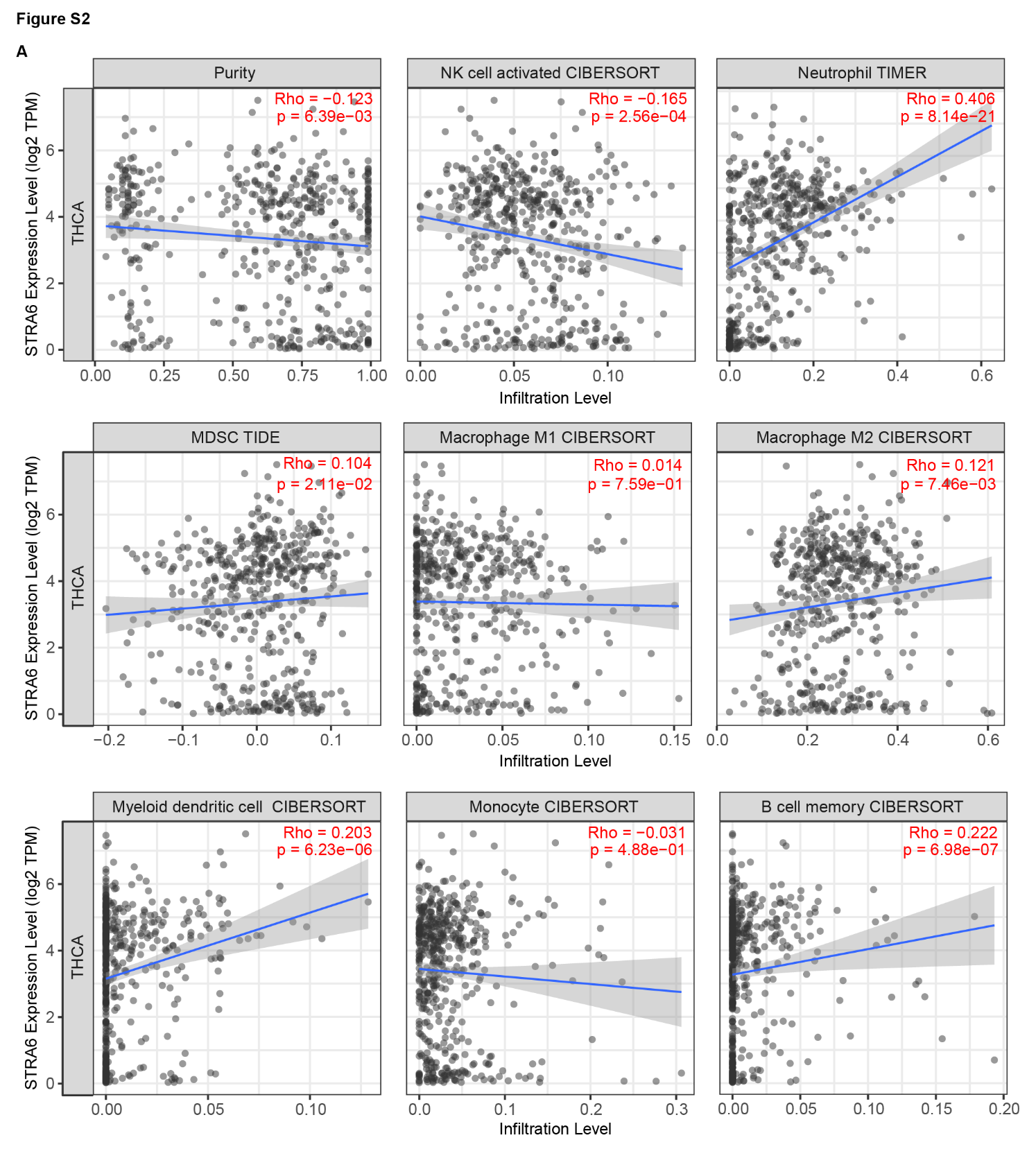


**Supplementary Figure 2.** STRA6 is correlated with immune cell infiltration. **(A)** Association of STRA6 expression with various immune cells (NK cells, neutrophils, MDSCs, M1, M2, myeloid dendritic cells, monocytes and B cells) infiltration estimated by Timer2.0.


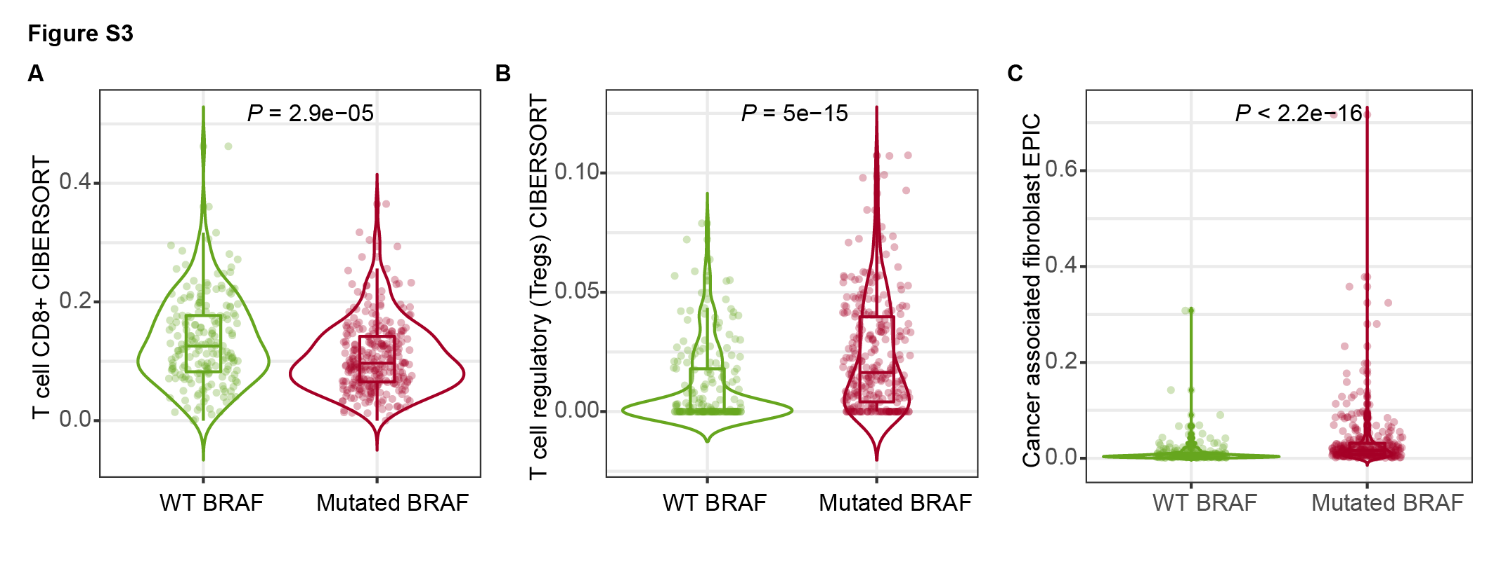
 **Supplementary Figure 3.** Immune cell infiltration level in PTC based on BRAF genotypes. **(A-C)** Infiltration of CD8+ T cells, Treg cells and CAFs in PTC according to BRAF mutation status.
